# DoE-based medium optimization for improved biosurfactant production with *Aureobasidium pullulans*

**DOI:** 10.3389/fbioe.2024.1379707

**Published:** 2024-03-06

**Authors:** Frederick Haala, Marie R. E. Dielentheis-Frenken, Friedrich M. Brandt, Tobias Karmainski, Lars M. Blank, Till Tiso

**Affiliations:** Institute of Applied Microbiology, RWTH Aachen University, Aachen, Germany

**Keywords:** polyol lipid, liamocin, exophilin, *Aureobasidium pullulans*, design of experiments, medium optimization, bioreactor, glycolipid

## Abstract

Polyol lipids (a.k.a. liamocins) produced by the polyextremotolerant, yeast-like fungus *Aureobasidium pullulans* are amphiphilic molecules with high potential to serve as biosurfactants. So far, cultivations of *A. pullulans* have been performed in media with complex components, which complicates further process optimization due to their undefined composition. In this study, we developed and optimized a minimal medium, focusing on biosurfactant production. Firstly, we replaced yeast extract and peptone in the best-performing polyol lipid production medium to date with a vitamin solution, a trace-element solution, and a nitrogen source. We employed a design of experiments approach with a factor screening using a two-level-factorial design, followed by a central composite design. The polyol lipid titer was increased by 56% to 48 g L^−1^, and the space-time yield from 0.13 to 0.20 g L^−1^ h^−1^ in microtiter plate cultivations. This was followed by a successful transfer to a 1 L bioreactor, reaching a polyol lipid concentration of 41 g L^−1^. The final minimal medium allows the investigation of alternative carbon sources and the metabolic pathways involved, to pinpoint targets for genetic modifications. The results are discussed in the context of the industrial applicability of this robust and versatile fungus.

## 1 Introduction

In the circular bioeconomy, biomass, waste, and CO_2_ (with green hydrogen) are utilized as carbon and energy sources for valuable products (Clomburg et al., 2017; Blank et al., 2020; Stegmann et al., 2020). Besides fossil fuels (oil demand above 12 million tons per day) (IEA, 2023), which will be replaced by renewable energy carriers, other volume-wise large chemical products are plastics (above 450 million tons per year) (Ritchie et al., 2023), organic solvents, and surfactants. The latter are produced from fossil resources or plant oils (e.g., palm oil). The alternative, for a sustainable and greenhouse gas reduced or even neutral production, are biosurfactants, surface-active molecules produced by microorganisms from renewable carbon sources (Irorere et al., 2017).

Biosurfactants are characterized by their microbial origin and chemical structure (Banat et al., 2010). The latter consists of a hydrophilic moiety, based on acids, peptide cations, or anions, mono-, di- or polysaccharides, and a hydrophobic moiety, based on saturated or unsaturated hydrocarbon chains or fatty acids (Banat et al., 2010), and hence is amphiphilic. Compared to synthetic or petrochemically derived surfactants, biosurfactants are readily biodegradable and often have low toxicity (Johann et al., 2016), rendering them an environmentally friendly alternative (Banat et al., 2010).

Due to their surface tension reducing properties in combination with high product-to-substrate yields (Y_P/S_) and a possible production from renewable feedstocks, glycolipids are currently the most industrially relevant group of biosurfactants (Paulino et al., 2016). The glycolipids’ hydrophilic moiety consists of a carbohydrate such as glucose, mannose, or rhamnose and is called glycone; the non-sugar component is called aglycone. Aglycones consist of saturated or unsaturated fatty acids, or hydroxy fatty acids and form a hydrophobic moiety (Henkel and Hausmann, 2019).

One group of well-studied glycolipids are rhamnolipids (RLs). Reaching industrial relevant titers of nearly 125 g L^−1^, the best characterized RL producer is *Pseudomonas aeruginosa* (Invally and Ju, 2020), whose human pathogenicity makes industrial use difficult. The recombinant RL producer *Pseudomonas putida* KT2440 does not have this disadvantage (Wittgens et al., 2011; Tiso et al., 2016; Soberón-Chávez et al., 2021). Due to their surface tension-reducing properties in aqueous solutions with a minimal value at around 20 mN m^−1^, RLs find application as detergents, emulsifiers, or foaming and dispersion agents (Xia et al., 2011; Wittgens and Rosenau, 2020; Guzmán et al., 2024). Evonik Industries AG with Unilever plc announced market entry in household cleaning products (Evonik Industries AG, 2020), while the first full industrial plant went online in January 2024 (Evonik Industries AG, 2024).

Other industrial available biosurfactants are sophorolipids, which consist of a sophorose glycone and a C_16_ or C_18_ fatty acid tail (Paulino et al., 2016). Depending on the composition, their use can reduce the surface tension between 30 and 50 mN m^−1^ (Roelants et al., 2019). Currently, sophorolipids find industrial applications in dishwashing liquids and personal care products (Evonik Industries AG, 2016). Reaching titers of more than 400 g L^−1^, the best producer is *Starmerella bombicola* (Daniel et al., 1998). Depending on fermentation strategy and strain, Y_P/S_ in the range of 0.3–0.7 g g^−1^ and volumetric productivity of up to 3.7 g L^−1^ h^−1^ are achieved (Roelants et al., 2019).

The third well-known glycolipids are mannosylerythriol lipids (MELs). MELs are produced by *Ustilago* sp. and *Pseudozyma* sp. (Arutchelvi et al., 2008; da Silva et al., 2021). With the latter, Rau et al. (2005) achieved titers of 165 g L^−1^, volumetric productivity of 13.9 g L^−1^ d^−1^ and an astonishingly high Y_P/S_ of 0.92 g g^−1^ using glucose and soybean oil as carbon source. Depending on the mixture, MELs reduce surface tension below 34 mN m^−1^ (Morita et al., 2013). Possible applications can be found in the food, cosmetics, and pharmaceutical industries, such as skin moisturizers (Morita et al., 2013; Morita et al., 2015; da Silva et al., 2021).

*Aureobasidium pullulans* is a yeast-like fungus that belongs to the division of ascomycetes (Schoch et al., 2006). Due to their ability to exist in various extreme habitats, *Aureobasidium* spp. are considered polyextremotolerant and not surprisingly occur ubiquitously (Prasongsuk et al., 2018; Gostinčar et al., 2019). In addition to their native robustness, *A. pullulans* strains can produce various enzymes and other products. Secreted xylanases, lipases, cellulases, amylases, mannanases, and laccases allow the metabolization of different carbon sources like hemicellulose, lignin breakdown products, and agricultural biomass (Nagata et al., 1993; Leathers et al., 2016; Prasongsuk et al., 2018; Zhang et al., 2019). One of the many potential carbon sources is sucrose, a disaccharide consisting of the monosaccharides glucose (G) and fructose (F). During metabolization of sucrose, enzymes with transfructosylating activity lead to the formation of fructooligosaccharides (FOS), each with one glucose monomer and up to four fructose units: 1-kestose (GF_2_), nystose (GF_3_), and fructofuranosylnystose (GF_4_) (Sánchez-Martínez et al., 2020; Liang et al., 2021). Due to their low calorific value, no carcinogenicity, and safety for diabetics, FOS are potential sweeteners in the food industry (Bali et al., 2015; Sánchez-Martínez et al., 2020).

Another valuable product from oleaginous *Aureobasidium* spp. are storage molecules, which are present as intracellular lipids, primarily as C_16:n_ or C_18:n_, and can reach 65% of the cell dry weight (Xue et al., 2018). Potential applications are in the biodiesel production (Liu et al., 2014; Sitepu et al., 2014; Song et al., 2022) or to replace plant oils as a resource for the polymer industry (Carlsson et al., 2011; Montero De Espinosa and Meier, 2011; Malani et al., 2022; Mangal et al., 2023). Palmitic acid (C_16:0_), oleic acid (C_18:1_), and linoleic acid (C_18:2_) are the primary fatty acids contained in oil crops (e.g., canola, palm, soybean, and sunflower) (Montero De Espinosa and Meier, 2011). The cultivation of these oil plants is the subject of much discussion regarding ecological and sociological sustainability (Meijaard et al., 2020). In addition, the fields compete with food production.

Polymalate is a secondary metabolite produced by *A. pullulans*. A polymer consisting of L-malic acid monomers that can potentially be used in drug delivery or tissue scaffolding (Portilla-Arias et al., 2010; Qiang et al., 2012; Kövilein et al., 2020). *A. pullulans* also belongs to the melanin-producing fungi and is thus considered a black yeast (Campana et al., 2022). The black pigment melanin is mainly produced after longer cultivation periods. It protects the organism from stress conditions such as high temperatures, high salt concentrations, ultraviolet radiation, oxidizing agents, or ionizing radiation (Gessler et al., 2014; Jiang et al., 2016). In Industry, *A. pullulans* is used to produce the polysaccharide pullulan, which consists of maltotriose units and finds applications in the food and pharma industry (Cheng et al., 2011; Sugumaran and Ponnusami, 2017).

**FIGURE 1:**
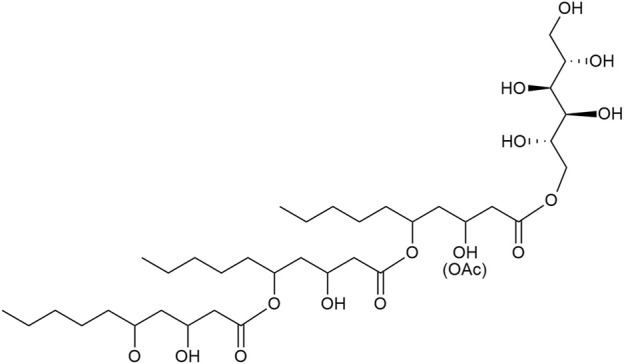
Representative chemical structure of a polyol lipid. Hydrophilic polyol headgroup (mannitol) with three 3,5-dihydroxy decanoic esters. Polyol lipids occur as congeners varying in the headgroup and the number of 3,5-dihydroxy decanoic esters.

The biosurfactants produced by *Aureobasidium* spp. are discussed for applications. They consist of polyol lipids a.k.a. liamocins and the aglycone oligo-dihydroxydecanoic acids (DDA), previously known as exophilins (Tiso et al., 2024) (Figure 1). They were first described as heavy oils (Nagata et al., 1993; Kurosawa et al., 1994) until Price et al. (2013) elucidated the molecular structure and named them liamocins. Polyol lipids are amphiphilic molecules consisting of a polyol headgroup (mainly mannitol, arabitol, or glycerol) with three or four esterified 3,5-dihydroxydecanoic acid groups, which can be acetylated at its 3-OH group (Leathers et al., 2013; Price et al., 2013; Manitchotpisit et al., 2014; Price et al., 2017). Molecules lacking the polyol headgroup were called exophilin, but were recently proposed to be renamed to oligo-dihydroxydecanoic acids (DDA) (Tiso et al., 2024). The congener distribution depends on strain, carbon source, and cultivation conditions (Price et al., 2017; Saika et al., 2020). Saur et al. (2019) showed that mainly polyol lipids with a mannitol headgroup (over 80%) and the aglycone DDA (over 15%) are produced. Polyol lipids are not yet produced industrially, but due to their amphiphilic properties, they show surface activity and reduce the surface tension between water and air to about 30 mN m^−1^ and can thus be used as a biosurfactant (Manitchotpisit et al., 2011; Kim et al., 2015). There are several hypotheses for the physiological role of liamocin. Due to the antimicrobial effect, the secreted polyol lipids could offer a competitive advantage over other microorganisms (Garay et al., 2018). Furthermore, increased lipase activity was observed at later cultivation times, suggesting that targeted digestion of the DDAs ensures carbon availability (Manitchotpisit et al., 2011; Leathers et al., 2013; Garay et al., 2018). Liamocin biosynthesis is not yet completely elucidated. It is proposed that acetyl- and malonyl-CoA are used by a polyketide synthase activated by a phosphopantetheinyl transferase to synthesize 3,5-dihydroxydecanoyl tails (Kim et al., 2015; Kang et al., 2021). Esterification of polyol headgroup and hydrophobic tail might be realized by esterase Est1 (Kang et al., 2021). In the following, the term polyol lipid refers to the natural mixture consisting of the polyol lipid and its aglycone DDA.

So far, cultivations for polyol lipid production with *A. pullulans* have been carried out in media containing complex components such as peptone or yeast extract. One of these media was described by Manitchotpisit et al. (2011) and optimized by Leathers et al. (2018) using statistical methods (Plackett-Burman design) and *A. pullulans* NRRL50384. This optimization doubled the titer up to 22 g L^−1^ and is the highest titer for an *Aureobasidium* spp. wild type achieved so far with a calculated Y_P/S_ of 0.18 g g^−1^. Zhang et al. (2022) reached the highest titer to date in a 10 L batch fermentation with 55 g L^−1^ and a Y_P/S_ of 0.47 g g^−1^ from glucose using a metabolically engineered *A. melanogenum* strain.

Currently, the biggest challenge is still the economic profitability of fermentative biosurfactant production. This obstacle can be overcome by increasing the productivity of strain and process and by reducing substrate and product purification costs (Sharma and Singh Oberoi, 2017; Roelants et al., 2019). Biosurfactants’ production costs depend heavily on substrate and raw materials (Akbari et al., 2023). However, 60%–80% of the costs of production are estimated for downstream processing (DSP) (Banat et al., 2014). Reducing raw material and DSP costs would have an enormous impact on production costs and, therefore, facilitate commercialization.

The first step to overcome these challenges is medium development and optimization, aiming for the best possible titer and Y_P/S_ from the utilized substrates. Basically, for growth and the synthesis of valuable products, microorganisms need a carbon and nitrogen source, minerals, vitamins, growth factors, oxygen, and water. The composition and interaction of these components are as crucial as the components themselves. In order to investigate these interactions, it is first necessary to develop a defined medium without complex components that may fluctuate from batch to batch. Besides medium composition, the amount of the components can influence growth and production, too, and is, therefore, a well-known optimization parameter. Design of experiments (DoE) uses statistical methods for planning, conducting, and analyzing experiments by varying the system influencing factors (here, media components) and investigating its effects on determined responses (here, titers and Y_P/S_) (Durakovic, 2017). DoE is an efficient tool to investigate interactions between production determining parameters, which is impossible with the popular but time-consuming one-factor-at-a-time method.

In this work, complex components of an existing medium were replaced with a defined nitrogen source, vitamin-, and trace element solution. Subsequently, the influence of particular media components on the polyol lipid titer was investigated using a two-level factorial design, and the medium was optimized using a central composite design. Finally, cultivations with the medium before and after optimization were transferred successfully from microtiter plates into 1 L bioreactor scale with a scaling factor of 350.

## 2 Material and methods

### 2.1 Strain

All cultivations were performed with *Aureobasidium pullulans* NRRL 62042 (ARS Culture Collection of the United States Department of Agriculture, Washington D.C., United States). This strain was isolated from a leaf in Thailand (Patalung) (Manitchotpisit et al., 2014).

### 2.2 Medium development

Previous works have produced polyol lipid in media that contain complex compounds. The complex medium optimized by Leathers et al. (2018) is the starting point for developing a minimal medium and consists of 1.5 g L^−1^ peptone, 0.9 g L^−1^ yeast extract, 1 g L^−1^ NaCl, 1 g L^−1^ K_2_HPO_4_, 0.8 g L^−1^ MgSO_4_ × 7 H_2_O, and 120 g L^−1^ sucrose. Yeast extract and peptone are replaced with a defined nitrogen source, a trace element, and a vitamin solution. The last two mentioned were previously used by Geiser et al. (2016) for cultivations with *Ustilago maydis*. The nitrogen content in yeast extract (0.13% w/w) and peptone (0.11% w/w) was estimated based on literature data (Garay et al., 2018) and replaced with 2.01 g L^−1^ NaNO3, 1.56 g L^−1^ (NH_4_)_2_SO_4_, 1.27 g L^−1^ NH_4_Cl, or 0.95 g L^−1^ NH_4_NO_3_. The vitamin solution contained per liter 0.05 g of D-biotin, 1 g of D-calcium pantothenate, 1 g of nicotinic acid, 25 g of myo-inositol, 1 g of thiamine hydrochloride, 1 g of pyridoxol hydrochloride, and 0.2 g of para-aminobenzoic acid. The trace element solution contained per liter 1.5 g of EDTA, 0.45 g of ZnSO_4_ × 7 H_2_O, 0.10 g of MnCl_2_ × 4 H_2_O, 0.03 g of CoCl_2_ × 6 H_2_O, 0.03 g of CuSO_4_ × 5 H_2_O, 0.04 g of Na_2_MoO_4_ × 2 H_2_O, 0.45 g of CaCl_2_ × 2 H_2_O, 0.3 g of FeSO_4_ × 7 H_2_O, 0.10 g of H_3_BO_3_, and 0.01 g of KI. Sucrose is replaced with glucose because *A*. *pullulans* can form FOS, which complicates substrate depletion analysis. To avoid MgNH_4_PO_4_ × 6 H_2_O formation, also known as struvite (Steimann et al., 2023), during medium preparation, MgSO_4_ × 7 H_2_O is added directly to the cultivation before inoculation to avoid precipitation.

### 2.3 Design of experiments

After successfully establishing a minimal medium based on the medium from Leathers et al. (2018) the developed medium was optimized by a design of experiments (DoE) approach. This method uses the advancement of statistical techniques to increase efficiency by reducing the amount of work and incorporating the dependence of components on each other. The latter does not take place in the often-used one-factor-at-a-time method. In this case, only one factor is changed simultaneously without considering their interaction. Experiments were planned and evaluated with the software DesignExpert11 (Stat-Ease Inc., Minneapolis, Minnesota, United States).

#### 2.3.1 Two-level factorial design

Since not all factors (media components) are likely to be involved in polyol lipid production, significant factors were identified with a two-level-factorial design to eliminate not-contributing factors from the study. The influence of each factor was determined by varying the concentration. One higher and one lower concentration was used, based on the medium from nitrogen source screening. All media components were selected as factors. Since Garay et al. (2018) summarized that the C/N (g_carbon_ per g_nitrogen_) ratio influences polyol lipid production, this was added as a factor and was adjusted via the ammonium nitrate quantity. The polyol lipid titer after 10 days of cultivation was selected as the response. Chosen high and low concentrations and the resulting center point are displayed in Table 1. Media compositions of the single experiments are shown in Supplementary Table S1.

**TABLE 1:** List of factors that are used in two-level factorial design: Glucose, carbon-to-nitrogen (C/N) ratio, K_2_HPO_4_, MgSO_4_ **×** 7 H_2_O, NaCl, trace element (TE) solution, and vitamin (vit.) solution were selected as factors. Based on the given low and high concentrations, the center point was determined and the experimental plan was designed.

Factor	Unit	Low concentration	Center point	High concentration
Glucose	g L^−1^	50	100	150
C/N ratio	g g^−1^	50	125	200
K_2_HPO_4_	g L^−1^	0.5	1.25	2
MgSO_4_ × 7 H_2_O	g L^−1^	0.5	1.25	2
NaCl	g L^−1^	0.5	1.25	2
TE-Solution	mL L^−1^	5	10	15
Vit.-Solution	mL L^−1^	0.5	1.25	2

#### 2.3.2 Response surface methodology—central composite design

After identifying factors significantly influencing the polyol lipid titer in the two-level factorial design, a central composite design was performed. All other factors were kept constant according to the center point of the two-level factorial design. A central composite design (CCD) consists of three components: In the factorial design, the factors are examined at two levels—high and low concentrations. The center points, where the mean values of the selected factors are repeated in an increased number of replicates to make the experiment more accurate. In addition, the star points are identical to the center points, but one factor is higher than the concentration of the factors used. The polyol lipid titer and Y_P/S_ were selected as responses after 10 days of cultivation. Media compositions of the single experiments are shown in Supplementary Table S3.

### 2.4 Cultivation conditions

#### 2.4.1 Preculture

From cryocultures, *Aureobasidium pullulans* NRRL 62042 was streaked out and incubated for 2 days at 30°C on yeast extract peptone agar plates (YEP, 20 g L^−1^ glucose, 20 g L^−1^ peptone, 10 g L^−1^ yeast extract, and 20 g L^−1^ agar). Precultures were inoculated with a single colony and cultivated at 30°C, with 300 rpm, and a shaking diameter of 50 mm for 22 h in YEP medium (20 g L^−1^ glucose, 20 g L^−1^ peptone, and 10 g L^−1^ yeast extract). Before inoculation, the culture broth was centrifuged (5 min, 17,000 × g), and the cell pellet was washed with ultrapure H_2_O.

#### 2.4.2 GrowthProfiler cultivations

Cultivations for the two-level factorial design were performed in 24-deep well microtiter plates (MTPs) (CR1424d, Enzyscreen BV, Heemstede, Netherlands) with a filling volume of 2 mL for 10 days at 30°C and 225 rpm with a shaking diameter of 25 mm using a GrowthProfiler (Enzyscreen BV, Heemstede, Netherlands). Cultures were inoculated to an OD_600_ of 0.2.

#### 2.4.3 SystemDuetz cultivations

The nitrogen and carbon source testing and experiments concerning the central composite design were performed in MTPs (CR1424a, Enzyscreen BV, Heemstede, Netherlands) with a filling volume of 2 mL for 10 days at 30°C and 300 rpm with a shaking diameter of 50 mm using a CR 1801h clamp system (Enzyscreen BV, Heemstede, Netherlands). Cultures were inoculated to an OD_600_ of 0.2.

#### 2.4.4 Bioreactor cultivation

A BioFlo120 bioreactor fermentation control unit from Eppendorf SE (Hamburg, Germany) was used for polyol lipid production in a glass bioreactor. It was equipped with a Pt100 temperature sensor, a pH probe (Bonaduz AG, EASYFerm Plus PHI 225, Hamilton Switzerland), dissolved oxygen (DO) probe (InPro6830, Mettler-Toledo, United States, Ohio Columbus), and BlueVary Offgas sensors (BlueSens gas sensor GmbH, Germany). The culture was inoculated to an OD_600_ of 0.5 and the cultivation was carried out at 30°C for 10 days. The DO was controlled at 30% by a stirrer cascade (300-1,000 min^−1^). Air was supplied with 0.5 vvm (volume air per volume medium per minute). 700 mL medium was used as working volume at a total volume of 1 L.

### 2.5 Polyol lipid determination

For polyol lipid determination, culture broth was centrifuged (5 min, 17,000 ×g). The cell pellet and polyol lipid were resuspended in acetonitrile. After another centrifugation step (5 min, 17,000 ×g), the acetonitrile, including dissolved polyol lipid, is separated from the cell fragments. The determination is carried out gravimetrically by evaporating the acetonitrile in pre-weighed glass vials using a speed vac at medium temperature (Savant Instruments, Speed Vac SC-100, Farmingdale, New York, United States). All wells from cultivations in MTPs undergo complete processing, followed by an additional acetonitrile rinse. After rinsing, the acetonitrile was used to resuspend the cell pellet, and polyol lipid after the first centrifugation step. In the case of samples obtained from the bioreactor, 1 mL of culture broth is utilized for each sample, with an accompanying 1 mL of acetonitrile.

### 2.6 Cell dry weight determination

For cell dry weight (CDW) determination, culture broth was centrifuged (5 min, 17,000 ×g). The cell pellet and polyol lipid were resuspended in 50% ethanol (v_EtOH_/v_H2O_). After another centrifugation step (5 min, 17,000 × g), the supernatant, including dissolved polyol lipid, was discarded. The cell pellet was resuspended in ultrapure H_2_O and transferred into pre-weighed glass vials. The determination is carried out gravimetrically by evaporating the H_2_O for 2 days at 70°C. A correction factor of 1.52 was determined by the cell damage caused by ethanol. Therefore, a YEP preculture was cultivated for 12 h, the culture broth centrifuged (5 min, 17,000 × g), and the cell pellet resuspended with ultrapure H_2_O or 50% ethanol (v_EtOH_/v_H2O_). All wells from cultivations in MTPs undergo complete processing, followed by an additional rinse with 50% ethanol. After rinsing, the 50% ethanol was used to resuspend the cell pellet and polyol lipid after the first centrifugation step. In the case of samples obtained from the bioreactor, 1 mL of culture broth is utilized for each sample, with an accompanying 1 mL of 50% ethanol (v_EtOH_/v_H2O_).

### 2.7 Pullulan determination

To determine pullulan concentrations, the culture broth was centrifuged (5 min, 17,000 × g), and the supernatant was mixed 1:1 (v/v) with ethanol. Precipitated pullulan was transferred after centrifugation (5 min, 17,000 × g) to a pre-weighed vial using ultrapure H_2_O. The determination is carried out gravimetrically after drying at 70°C for 2 days.

### 2.8 Glucose and fructooligosaccharide determination

Glucose, fructose, sucrose, 1-kestose, nystose, and fructofuranosylnystose were determined by high-performance liquid chromatography (HPLC). After centrifugation (5 min, 17,000 × g) of 1 mL culture broth, the supernatant was mixed 1:1 (v/v) with ethanol to precipitate pullulan. Samples were analyzed using an UltiMate 3000 HPLC System (Thermo Fisher Scientific, Waltham, Massachusetts, United States), including an Ultimate 3000 pump, Ultimate 3000 autosampler, Ultimate 3000 column oven (Thermo Fisher Scientific, Waltham, Massachusetts, United States) and a Knauer RI-Detector RefractoMax 521 (KNAUER Wissenschaftliche Geräte GmbH, Berlin, Germany). When glucose was used as a carbon source, substrate consumption was determined with a Metab-AAC column (BF-series, Ion exchange, 300 × 7.8 mm) from Isera GmbH (Düren, Germany) at 40 °C and a flow rate of 0.6 mL min^-1^. The mobile phase consisted of 5 mM H_2_SO_4_. Using sucrose as a carbon source, glucose, fructose, and FOS concentration were measured with a NUCLEODUR HILIC column (Multospher APS HP–3 µm HILIC, 250 × 2 mm) from CS-Chromatographie Service GmbH (Langerwehe, Germany) at 50 °C and a flow rate of 0.5 mL min^-1^. The mobile phase consisted of 80% acetonitrile (v_ACN_/v_H2O_). Measurements were analyzed using Chromeleon 7.2.10 software (Thermo Fisher Scientific, Waltham, Massachusetts, United States).

## 3 Results

So far, polyol lipid production has been conducted in the literature in a medium with complex components and sucrose. After replacing sucrose with glucose to avoid fructooligosaccharide formation, a minimal medium was designed by replacing yeast extract and peptone with vitamins, trace elements, and a nitrogen source to substitute these undefined and expensive components. Several components were tested as nitrogen source. After medium development, a medium optimization using a design of experiments (DoE) approach was performed, followed by transferring the production cultivation from microtiter plate (MTP) to the bioreactor scale.

### 3.1 Simplifying quantification of substrate uptake kinetics by replacing sucrose with glucose

While using sucrose as carbon source, *A. pullulans* builds up and consumes fructooligosaccharides (FOS). FOS are polysaccharides consisting of one glucose and two or more fructose units. To streamline the process and simplify substrate uptake kinetics, sucrose is replaced by glucose as carbon source. To ensure that this does not lead to reduced production or impaired growth, the two carbon sources were compared with each other. For this purpose, cultivations with the same amount of carbon were performed in MTPs using SystemDuetz and the complex medium developed by Leathers et al. (2018).

**FIGURE 2:**
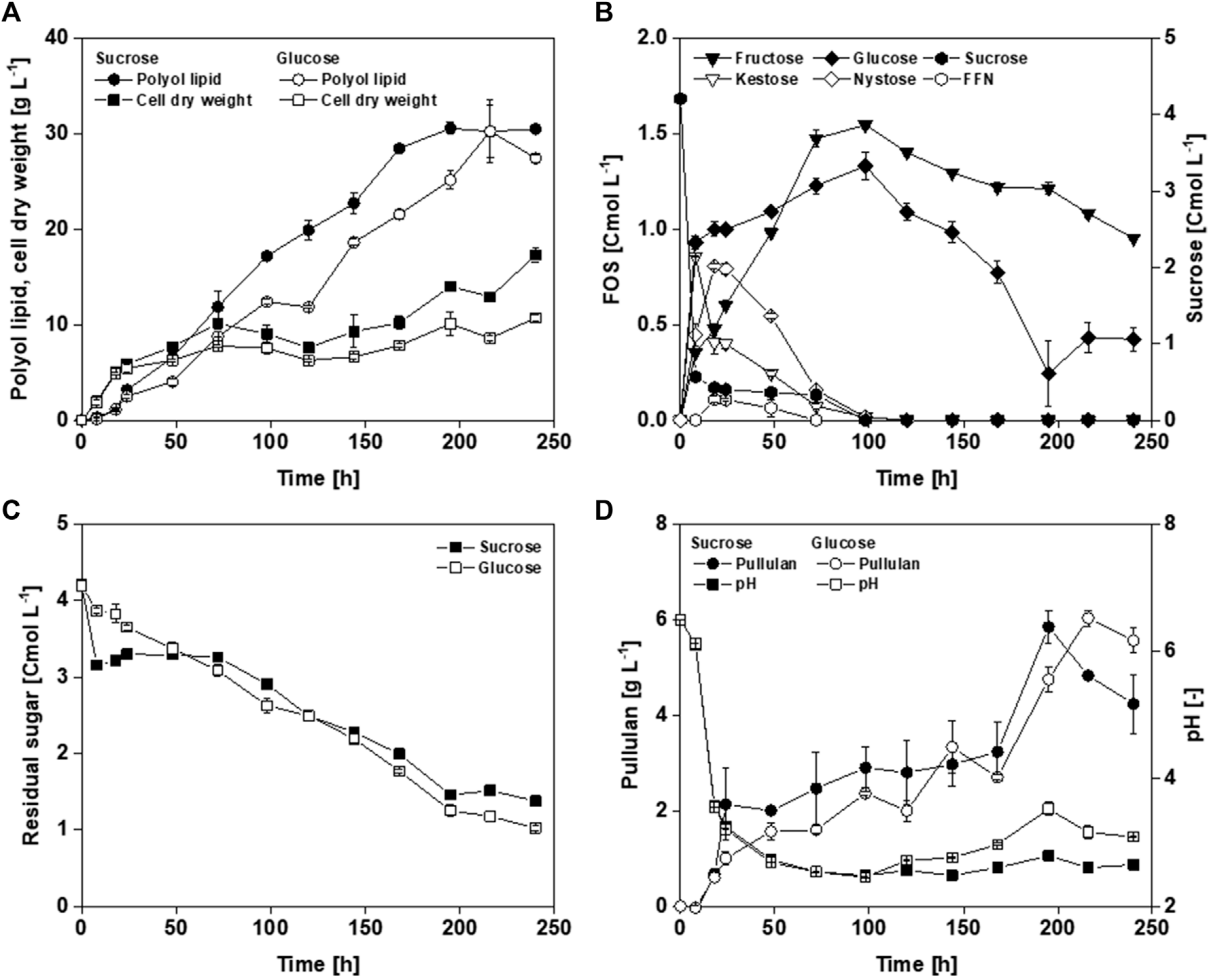
Cultivation of *A. pullulans* NRRL 62042 with different carbon sources: Comparison of cell dry weight **(A)**, polyol lipid **(A)**, fructooligosaccharides (FOS) (sucrose, kestose, nystose, and fructofuranosylnystose (FFN)) and their monomers glucose and fructose **(B)**, sum of the residual sugars **(C)**, pullulan **(D)**, and pH **(D)** over time by using different carbon sources (4.2 Cmol L^−1^ of glucose and sucrose). Cultivations were performed with *A. pullulans* NRRL 62042 in 24 deep-well microtiter plates with 2 mL filling volume, 30°C, and 300 rpm. Data show the mean of triplicates with ±standard deviation.

Within the first 24 h, exponential growth occurred until 5.5–6.0 g L^−1^ CDW (Figure 2A). After the exponential growth phase, the CDW increased to 17.3 g L^−1^ using sucrose and 10.7 g L^−1^ using glucose as a carbon source. This resulted in a final product-to-biomass yield (Y_P/X_) from sucrose and glucose of 1.8 g g^−1^ and 2.6 g g^−1^, respectively. Analyzing off-gas data in bioreactor cultivations (data not shown), a substrate limitation except carbon was found, which could be attributed to a nitrogen limitation between 24 and 48 h. As nitrogen is essential for cell growth, biomass stagnation occurs in a limitation. In the absence of nitrogen, a constant biomass concentration should, therefore, be measured until cell death. *A. pullulans* form intracellular storage lipids under unbalanced cultivation conditions, like high carbon (C) and low nitrogen (N) concentrations (high C/N ratio). These lipids are still present in the biomass when determining CDW and are thus included in the weight (Ratledge and Wynn, 2002; Xue et al., 2018). This increase in biomass, without increasing the number of active cells, leads to a lower Y_P/X_. With both carbon sources, maximal polyol lipid titers of approximately 30 g L^−1^ were achieved (Figure 2A). For this reason, it is assumed that growth ends after 48 h and Y_P/X_ are calculated using the measured biomasses at this time. This resulted in a Y_P/X_ from sucrose and glucose of 4.8 g g^−1^ and 4.0 g g^−1^, respectively. At the end of the cultivation, 0.42 Cmol L^−1^ glucose and 0.95 Cmol L^−1^ fructose remained when sucrose was used as a carbon source (Figure 2B). During cultivation, almost all sucrose was converted to glucose, fructose, 1-kestose, and nystose within the first 8 h. After that, the glucose concentration stagnated markedly, indicating that glucose was metabolized. The 1-kestose concentration decreased after 8 h, while fructose, nystose, and fructofuranosylnystose accumulated. After 100 h, all sugars were converted into the monomers fructose and glucose and were metabolized simultaneously. When glucose was used as the initial carbon source, 1.0 Cmol L^−1^ was left after 240 h (Figure 2C). Furthermore, Figure 2C shows that the consumption of the residual sugars is similar when glucose or sucrose is used as the initial carbon source. Substrate costs make up to 50% of the production costs (Akbari et al., 2023). Leftover substrate is discarded at the end of a batch process and thus lowers the economic efficiency. Therefore, the product-to-substrate yields (Y_P/S_) calculated in this work refer to the initial carbon amount. Y_P/S_ is given in gram product per Cmol substrate to compare glucose and sucrose. Using glucose as carbon source resulted in a higher Y_P/S_ of 7.7 g Cmol_glucose_^−1^ compared to 7.2 g Cmol_sucrose_^−1^. In both cultivations, approximately 6 g L^−1^ pullulan was produced, which appeared to be degraded toward the end (Figure 2D). Furthermore, the pH value in both cultivations decreased to approximately 3 within 24 h and remained below 3 (Figure 2D). This low pH value might be due to the production of PMA, whose malate monomers have a free acid group. Altogether, the only noticeable difference seems to be higher biomasses when using sucrose as carbon source after exponential growth, possibly due to the assumed increased formation of intracellular storage lipids using sucrose as carbon source. In addition, gravimetrical CDW determination after washing the cells with 50% ethanol could lead to certain deviations. Regarding growth behavior and polyol lipid production, *A. pullulans* NRRL 62042 did not show mentionable differences between glucose and sucrose. Having in mind that sucrose entails more complex carbon uptake kinetics, glucose was used in the following experiments.

### 3.2 Streamlining the medium: complex components could successfully be replaced

Previously used media included complex nitrogen sources such as yeast extract and peptone or complex carbon, and nitrogen sources like corn steep liquor, xylose mother liquor, or gluconate mother liquor (Cheng et al., 2017; Leathers et al., 2018; Li et al., 2021). For the development of a minimal medium, complex components have to be replaced. Therefore, several defined nitrogen sources were tested: NH_4_NO_3_, NaNO_3_, NH_4_Cl, and (NH_4_)_2_SO_4_. Yeast extract and peptone contain trace elements and vitamins; these were substituted using vitamin and trace-element solutions. Cultivations were performed in MTPs for 10 days in SystemDuetz.

**FIGURE 3:**
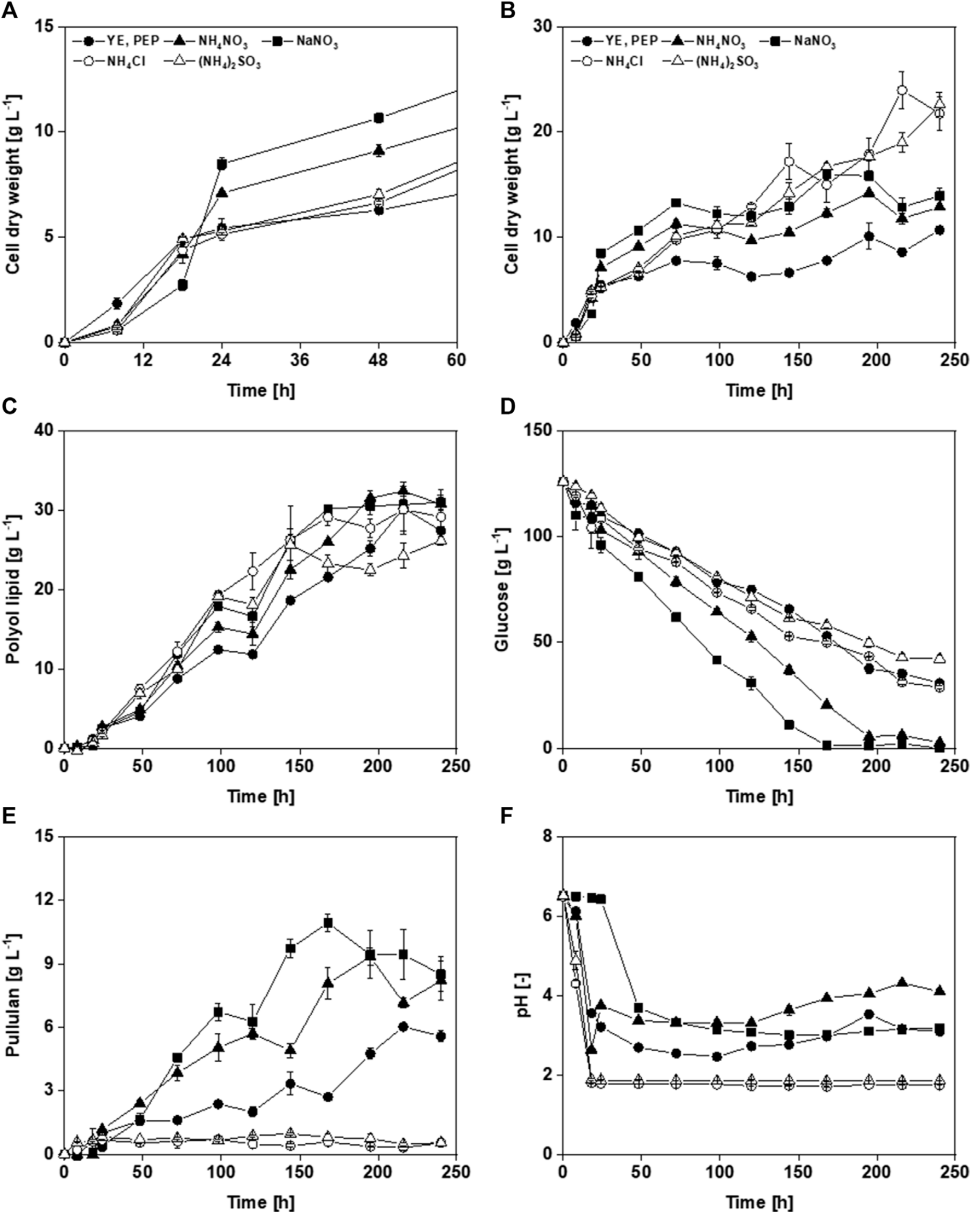
Cultivation of *A. pullulans* NRRL 62042 with different nitrogen sources: Comparison of cell dry weight over the first 48 h **(A)**, and 240 h **(B)**, polyol lipid **(C)**, glucose **(D)**, pullulan **(E)**, and pH **(F)** over time by using 126 g L^−1^ glucose and different nitrogen sources (0.9 g L^−1^ yeast extract (YE) and 1.5 g L^−1^ peptone (PEP), 2.01 g L^−1^ NaNO3, 1.56 g L^−1^ (NH_4_)_2_SO_4_, 1.27 g L^−1^ NH_4_Cl, or 0.95 g L^−1^ NH_4_NO_3_). Cultivations were performed with *A. pullulans* NRRL 62042 in 24 deep-well microtiter plates with 2 mL filling volume, 30°C, and 300 rpm. Data show the mean of triplicates with ±standard deviation.

Compared to the yeast extract- and peptone-containing medium, the media with defined nitrogen sources seemed to have a longer lag phase (Figure 3A), arguing for an adapted preculture preparation. When NaNO_3_ was used, the lag phase was 8 h longer. This is probably because microorganisms first convert nitrate into ammonium, which requires energy and thus results in slow growth (Siverio, 2002). Cultivations with media containing ammonium salts (NH_4_NO_3_, NH_4_Cl, and (NH_4_)_2_SO_4_) showed an almost identical growth phase. Strikingly, nitrogen sources containing nitrate resulted in higher CDW in the first 120 h (Figure 3B). After the exponential growth phase, the biomass concentration using yeast extract- and peptone-containing medium was 6.3 g L^−1^, comparable to those media with (NH_4_)_2_SO_4_ and NH_4_CI as nitrogen sources. With nitrate salts, CDWs from NH_4_NO_3_ and NaNO_3_ of 9.1 g L^−1^ and 10.7 g L^−1^, respectively, were reached after 48 h. This could be due to the increased intracellular storage lipid formation. Polyol lipid production was highest using NH_4_NO_3_ with a titer of 32.4 g L^−1^ (Figure 3C). However, taking into consideration the standard deviation, all cultivations are in a similar range concerning the maximal polyol lipid titers. NH_4_Cl-containing medium achieved the highest Y_P/X_ with 4.5 g g^−1^. The lowest was 2.9 g g^−1^ by using NaNO_3_. After the growth phase, glucose consumption was almost linear under all conditions (Figure 3D). In the cultivations where nitrate was used, glucose was depleted after 168 h (NaNO_3_) and 192 h (NH_4_NO_3_). The others still contained at least 28 g L^−1^ residual sugar, comparable to the reference with complex nitrogen sources. When glucose was depleted, pullulan concentrations reached their highest values with up to 11 g L^−1^, a concentration that started to decrease afterward (Figure 3E). With NH_4_Cl and (NH_4_)_2_SO_4_, almost no pullulan was produced (c_pullulan,max_ < 1 g L^−1^). Moreover, pullulan formation stagnated in these cultivations after the pH dropped below 2. When NH_4_NO_3_ was used as a nitrogen source, the pH value increased after 24 h (Figure 3F). This might be due to the diauxic metabolism of NH_4_^+^ and NO_3_^−^. While metabolizing the preferred NH_4_^+^ (base), the counter ion NO_3_^−^ (acid) remains in the culture broth, leading to its acidification (sharp drop in the pH value during the first 24 h). After NH_4_^+^ is depleted, metabolizing NO_3_^−^ removes the acid from the culture broth, which leads to alkalization (increase in pH value). The subsequent slow drop of the pH value is presumably associated with PMA production. Using nitrate-containing nitrogen sources tends to result in slightly higher pH values than pure ammonium-containing ones. Furthermore, dark coloration of the culture broth was observed after 48 h for the media containing NH_4_Cl and (NH_4_)_2_SO_4_, which changed to a deep black after 72 h, indicating melanin formation (Jiang et al., 2016).

Depending on the media composition, carbon remains present even after 10 days. The carbon consumption using the initial medium was not described by Leathers et al. (2018). A direct comparison is, therefore, not possible. It was also shown that the increased carbon consumption was due to increased pullulan formation. The strain *A. pullulans* NRRL50384 (equivalent to RSU29) used by Leathers et al. (2018) in their medium development is known as a strong pullulan producer, enabling the complete consumption of sucrose (Manitchotpisit et al., 2014). In cultivations in which lower pH values were reached, also low pullulan titers were obtained (Figure 3F). In batch fermentations with *A. pullulans* CCTCC 209298, where the pH was controlled, higher pullulan production was noted at pH 3.5 (26.3 g L^−1^) and 4.5 (24.6 g L^−1^), while the lowest titer (18.5 g L^−1^) was achieved at pH 2.5 (Wang et al., 2013).

Altogether, sucrose, yeast extract, and peptone were successfully replaced. Although NH_4_Cl and (NH_4_)_2_SO_4_ offer the advantage of preventing pullulan production by a low pH and resulted in a better Y_P/S_, using only NH_4_ as a nitrogen source led to melanin formation. Melanin may stain the product and thus complicate purification, while pullulan remains in the aqueous phase and can thus be easily separated from polyol lipid. Cultivation with NH_4_NO_3_ as a nitrogen source showed a shorter lag phase and lower pullulan production than NaNO_3_. The maximum polyol lipid titer and Y_P/S_ were comparable, while a higher Y_P/X_ was achieved using NH_4_NO_3_. Accordingly, NH_4_NO_3_ was selected as the nitrogen source for subsequent experiments.

### 3.3 Design of experiments—optimized medium for polyol lipid production

After developing a defined minimal liamocin production medium (MLP), a DoE approach was used for medium optimization regarding polyol lipid production. First, medium components were investigated to identify factors significantly influencing polyol lipid production. Therefore, a two-level factorial design was used. Second, the concentrations of factors influencing polyol lipid titer were optimized by a central composite design. The results were assessed by analysis of variance (ANOVA) and summarized in tabular form. In ANOVA, the significance of an observed effect is assessed using the *p*-value. A significant effect can be assumed if the *p*-value is less than 5% (*p* < 0.05). The smaller the *p*-value, the more important the effect. To investigate whether a scatter is due to an effect or to noise, the variance between the groups (prediction variance) is compared with the variance within the groups (error variance) by calculating the quotient of the sums of squares, yielding the F-value. A group is a concentration of a factor. There are, therefore, three groups per factor (component), each of which is defined by the concentration of the factor. The larger the F-value, the greater the scatter between the individual groups compared to the error variance. The greater the variance (F-value) between the groups, the more likely there are significant differences between them. The model is the mathematical representation of the relationships between the individual factors. The lack of fit describes the extent to which the model’s prediction misses the observations or measured values. Therefore, a non-significant lack of fit means that the model can predict the measured values.

#### 3.3.1 Two-level factorial design—glucose and phosphate significantly influence polyol lipid titers

A two-level factorial design was used to identify which medium components (factors) influence the polyol lipid titer. Therefore, factors were investigated using one high and one low concentration (Table 1). The individual experiments (runs) and corresponding polyol lipid titers (responses) are shown in Supplementary Table S1. As described previously, experiments were conducted in MTPs with a filling volume of 2 mL for 10 days at 30°C and 225 rpm.

The results were analyzed by ANOVA and are displayed in Table 2. The model’s F-value of 131.41 implies that the model is significant, with a 0.01% chance that an F-value this large could occur due to noise. *p*-values below 0.05 indicate that the model or factors are significant. Accordingly, glucose and the interaction between glucose and K_2_HPO_4_ (entry “AB” in Table 2) are factors that significantly influence polyol lipid titers. Factors with a *p*-value above 0.1 are not significant. With a *p*-value below 0.1 and above 0.05, K_2_HPO_4_ as a single factor will not improve the model but contribute to its hierarchy. The model is hierarchical if all lower-order terms (A), which assemble the higher-order terms (AB), are also contained in the model.

**TABLE 2:** ANOVA analysis from two-level factorial design: ANOVA analysis shows a significant (*p* < 0.05) effect on polyol lipid production for glucose and the interaction between glucose and K_2_HPO_4_.

Factor	Degrees of freedom	Mean square	F-value	*p*-value
Model	3	937.26	131.41	0.0001
K_2_HPO_4_ (A)	1	26.10	3.66	0.0647
Glucose (B)	1	2,712.16	380.26	0.0001
AB	1	73.51	10.31	0.0030
Lack of fit	29	7.70	4.77	0.1112

**FIGURE 4:**
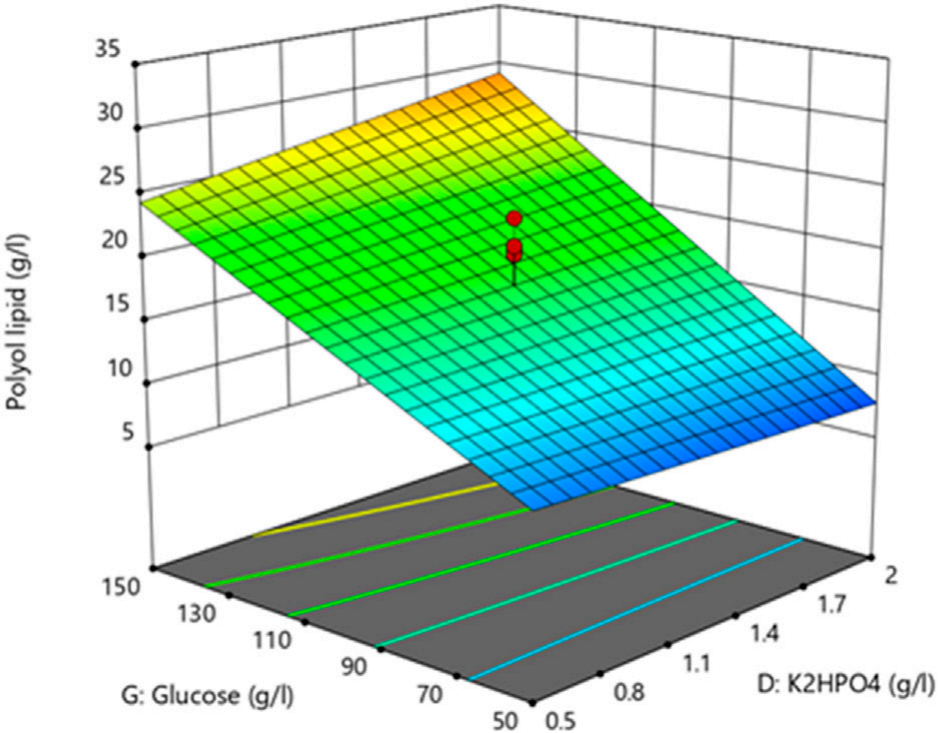
3D-surface diagram from two-level factorial design: Interaction between glucose and K_2_HPO_4_ concentration influences the polyol lipid titer. Increasing polyol lipid titers are displayed by a color gradient from low (blue) to high (orange). Center points are represented by red dots.

The lack of fit’s F-value with a *p*-value of 0.1112 implies an 11.12% chance that an F-value this large could occur due to noise. With a *p*-value above 0.1, the lack of fit is not significant, implying that the model fits. All other factors had no statistically significant impact (Supplementary Table S2). The resulting three-dimensional surface diagram is shown as a heat map in Figure 4 and displays the dependency of the polyol lipid titer on the glucose and K_2_HPO_4_ concentration. Rising glucose concentrations led to increasing polyol lipid titers, which were additionally increased by rising K_2_HPO_4_ concentrations, especially at glucose concentrations above 130 g L^−1^. This could indicate a phosphate limitation at high carbon concentrations, which will be investigated in later experiments. A maximum polyol lipid titer of 28 g L^−1^ was predicted using 150 g L^−1^ glucose and 2 g L^−1^ K_2_HPO_4_, which were the maximum concentrations included in the factor screening. Aiming a global and not a local maximum, glucose, and K_2_HPO_4_ concentrations were increased during optimization. All other media components were kept constant according to their center point concentrations.

#### 3.3.2 Central composite design—increasing yield and titer by optimizing glucose and phosphate content

The two-level factorial design revealed glucose and the interaction between K_2_HPO_4_ and glucose as significant factors for polyol lipid production in *A. pullulans* NRRL 62042. These two factors are used in the following central composite response surface design. The limits were set higher according to the result of the factor screening (Table 3). Experiments were carried out in random order (*n* = 5). The experiment in the central point was carried with an n of 8. All individual experiments and corresponding responses of polyol lipid titer and Y_P/S_ are shown in Supplementary Table S3.

**TABLE 3:** List of factors that are used in central composite design: After using a two-level factorial design to identify glucose and K_2_HPO_4_ as factors significantly influencing the polyol lipid titer, these were selected as factors in the central composite design. Based on the given low and high concentrations, the center and star points were determined and the experimental plan was designed.

Factor	Unit	Low conc	Center point	High conc	Star point max	Star point min
Glucose	g L^-1^	120	210	300	337.3	82.7
K_2_HPO_4_	g L^-1^	1.4	4.7	8.0	9.37	0.03

**FIGURE 5:**
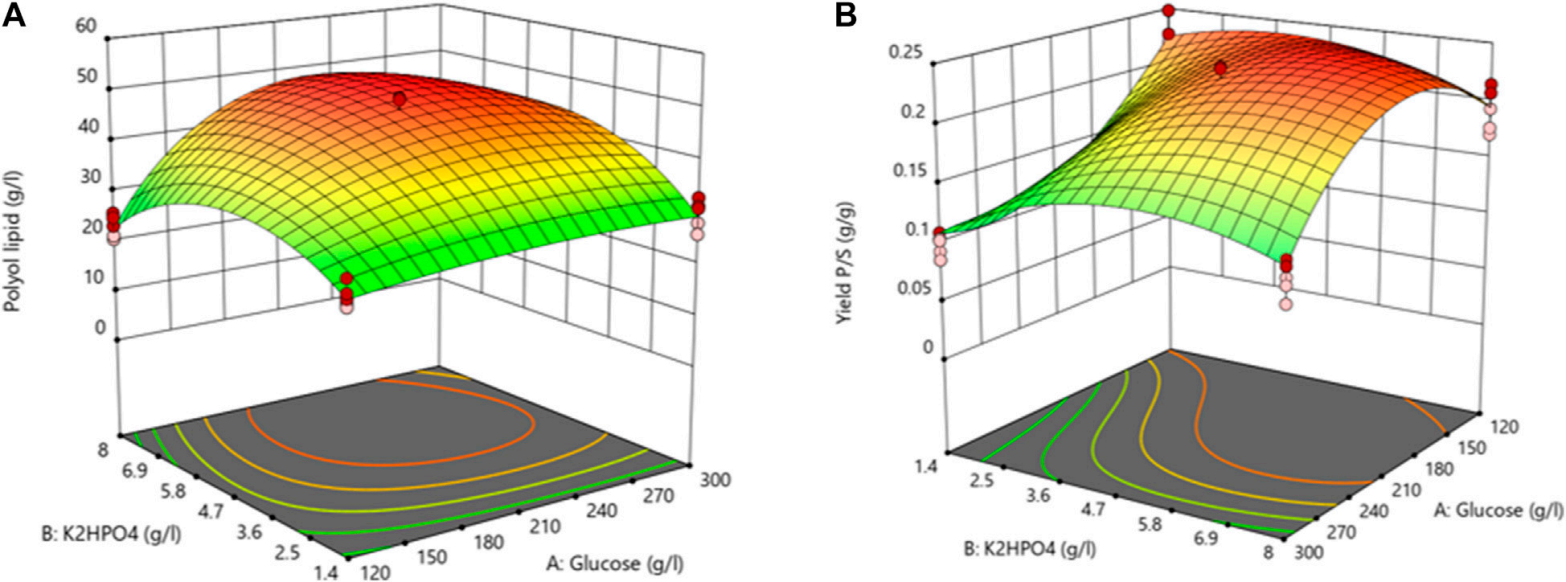
3D-surface diagram from central composite design: Interaction between glucose and K_2_HPO_4_ in the central composite design influencing the polyol lipid titer **(A)** and the product per substrate yield **(B)**. Increasing values are displayed by a color gradient from green (low) to red (high).

Table 4 summarizes the ANOVA results investigating polyol lipid titer as the response. Due to a *p*-value below 0.05, all factors and the model significantly influenced the polyol lipid titer. The lack of fit’s *p*-value between 0.05 and 0.1 is not significant nor optimal. There is a 9.82% chance that a lack of fit F-value this large could occur due to noise. The heat map resulting from the model with the polyol lipid titer as the response is shown in Figure 5A. Using 232.5 g L^−1^ glucose and 6.02 g L^−1^ K_2_HPO_4_ a maximum polyol lipid titer of 50.1 g L^−1^ was predicted and forms a global maximum, indicating that the design space has been hit. Higher or lower glucose or K_2_HPO_4_ concentrations led to lower polyol lipid titers.

**TABLE 4:** ANOVA analysis from central composite design with polyol lipid titer as response: ANOVA analysis indicates a significant (*p* < 0.05) effect on polyol lipid production for glucose (A), K_2_HPO_4_ (B), and the interaction between glucose and K_2_HPO_4_ (AB). The *R* ^2^ is 0.97.

Factor	Degrees of freedom	Mean square	F-value	*p*-value
Model	7	6.649 × 10^5^	157.25	< 0.0001
Glucose (A)	1	4.739 × 10^5^	112.06	< 0.0001
K_2_HPO_4_ (B)	1	1.675 × 10^5^	396.16	< 0.0001
AB	1	1.086 × 10^5^	25.68	< 0.0001
A^2^	1	1.673 × 10^5^	395.55	< 0.0001
B^2^	1	1.602 × 10^5^	378.86	< 0.0001
A^2^B	1	6.581 × 10^5^	155.64	< 0.0001
AB^2^	1	17560.27	4.15	0.0486
Lack of Fit	1	11594.58	2.88	0.0982

Due to a *p*-value below 0.05 (Table 5), all factors and the model itself showed a significant influence on Y_P/S_. With a *p*-value above 0.1, the lack of fit is not significant, implying that the model fits. A maximum Y_P/S_ of 0.25 g g^−1^ was predicted using 176.7 g L^−1^ glucose and 5.97 g L^−1^ K_2_HPO_4_ (Figure 5B). Further, the heat map shows that glucose concentrations above 210 g L^−1^ have a negative effect on Y_P/S_. This is because residual sugar was still present after the cultivation period of 10 days. High sugar concentrations also increase the osmotic pressure, which slows down growth. In comparison, the amount of K_2_HPO_4_ used had a less pronounced effect in the selected range.

**TABLE 5:** ANOVA analysis from central composite design with product-to-substrate yield as response: ANOVA analysis indicates a significant (*p* < 0.05) effect on the product-to-substrate yield for glucose (A), K_2_HPO_4_ (B), and the interaction between glucose and K_2_HPO_4_ (AB). The *R* ^2^ is 0.97.

Factor	Degrees of freedom	Mean square	F-value	*p*-value
Model	7	0.0090	171.93	< 0.0001
Glucose (A)	1	0.0273	524.59	< 0.0001
K_2_HPO_4_ (B)	1	0.0189	363.31	< 0.0001
AB	1	0.0013	24.80	< 0.0001
A^2^	1	0.0039	74.55	< 0.0001
B^2^	1	0.0095	182.82	< 0.0001
A^2^B	1	0.0108	206.49	< 0.0001
A^2^B^2^	1	0.0003	5.01	0.0311
Lack of Fit	1	0.0001	2.73	0.1067

For a maximum titer and Y_P/S_ the model predicted the optimized minimal polyol lipid production medium (oMLP) to consist per liter of 208 g glucose, 6.30 g K_2_HPO_4_, 1.91 g NH_4_NO_3_, 1.25 g MgSO_4_ × 7 H_2_O, 1.25 g NaCl, 1.25 mL vitamin solution, and 10 mL trace element solution. The resulting C/N ratio is 125 g_carbon_ per g_nitrogen_. With this composition, the model predicted a polyol lipid titer of 49.69 g L^−1^ and a Y_P/S_ of 0.23 g g^−1^ (Table 6). Cultivations for 10 days at 30 °C with 300 rpm in MTPs were used to confirm the model’s prediction. The achieved polyol lipid titer of 48.07 g L^−1^ and Y_P/S_ of 0.23 g g^−1^ were inside the confidence level (95%) of the prediction interval (PI). These results confirm the model predictions. Moreover, this is the highest achieved polyol lipid titer using an *A. pullulans* wildtype strain so far.

**TABLE 6:** Model confirmation after medium optimization: Data mean of polyol lipid and product-to-substrate yield Y_P/S_. Cultivations were performed in 24-well microtiter plates with 2 mL filling volume at 30°C and 300 rpm for 10 days (n = 3).

Factor	Unit	Predicted mean	Predicted median	Standard deviation	n	95% PI low	Data mean	95% PI high
Polyol lipid	g L^-1^	49.69	49.71	1.70	3	47.33	48.07	52.00
Y_P/S_	g g^-1^	0.24	0.24	0.01	3	0.23	0.23	0.25

#### 3.3.3 One-factor-at-a-time experiment—phosphate as a single factor has no considerable impact on polyol lipid production

**FIGURE 6:**
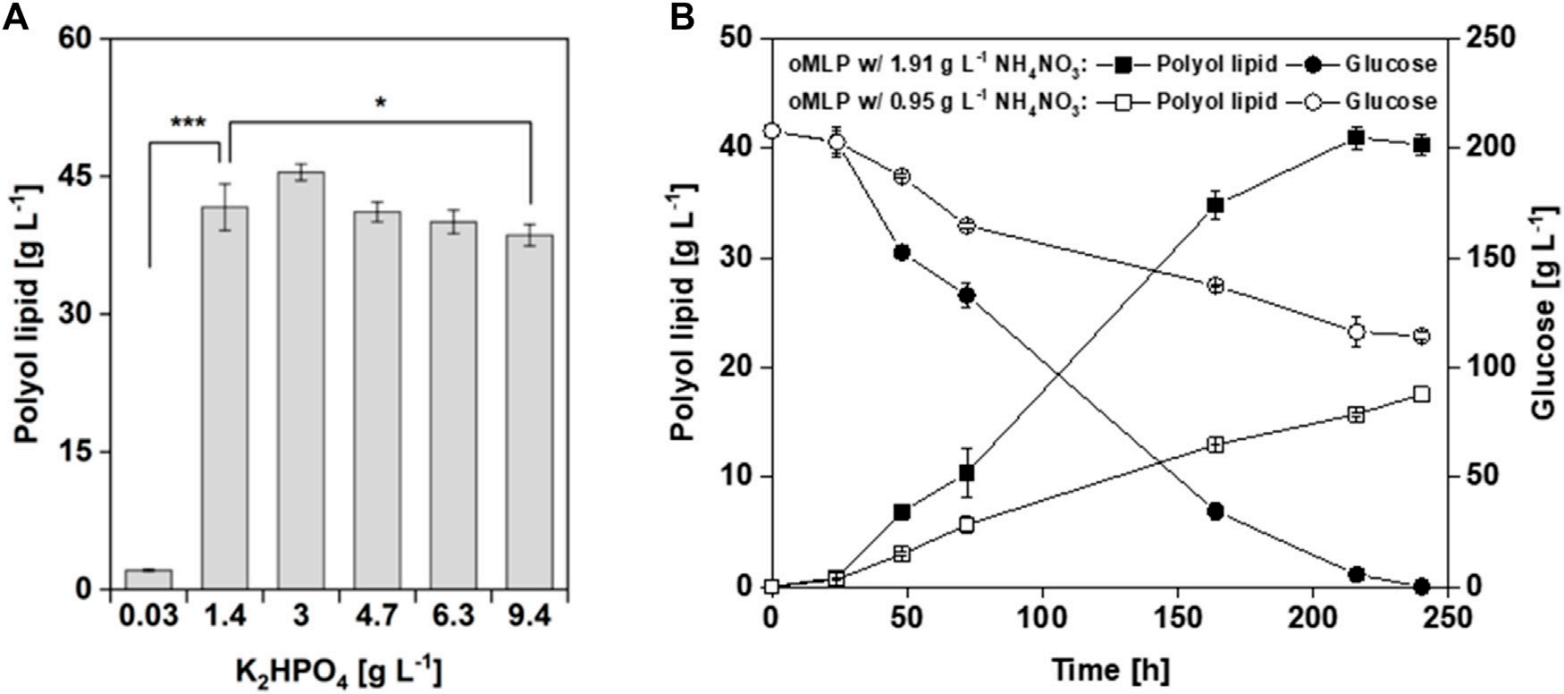
Cultivation of *A. pullulans* NRRL62042 with different phosphate and nitrogen concentrations: Polyol lipid production using the optimized MLP medium with different K_2_HPO_4_ concentrations after 240 h **(A)**. Polyol lipid production and glucose consumption over time using the optimized MLP medium with different NH_4_NO_3_ concentrations **(B)**. Cultivations were performed with *A. pullulans* NRRL 62042 in 24 deep-well microtiter plates with 2 mL filling volume, 30 °C, and 300 rpm. Data show the mean of triplicates with ±standard deviation. Statistically significant differences in polyol lipid production were determined by Welch’s *t*-test and are indicated as * (*p*-value ≤0.5) and *** (*p*-value ≤0.001).

In the two-level factorial design, factors (medium components) that significantly (*p* < 0.05) influence polyol lipid titer were investigated. As a result, besides glucose, the combined factor of glucose and K_2_HPO_4_ was identified as significantly influencing polyol lipid production, which suggests a phosphate limitation. K_2_HPO_4_ as a single factor had a *p*-value between 0.05 and 1 and is, therefore, per definition, neither significant nor insignificant. To investigate the influence of K_2_HPO_4_ as a single factor on polyol lipid production, a one-factor-at-a-time experiment was conducted for 10 days in MTPs using the optimized MLP medium and different K_2_HPO_4_ concentrations between 0.03 g L^−1^ and 9.36 g L^−1^. The results are shown in Figure 6A. The highest titer of 46.3 g L^−1^ was achieved using 3.0 g L^−1^ K_2_HPO_4_. With a K_2_HPO_4_ concentration of at least 1.4 g L^−1^, similar polyol lipid titers could be achieved, with a slight decrease using concentrations above 3.0 g L^−1^. A statistical analysis (Welch’s *t*-test) showed that there is no significant differences in the polyol lipid titers using K_2_HPO_4_ concentrations between 1.4 and 6.3 g L^−1^ Leathers et al. (2018) showed that none of the inorganic salts contained in their medium significantly influence polyol lipid production in the two-level factorial design. Only increasing K_2_HPO_4_ concentrations above 2.5 g L^−1^ inhibit polyol lipid formation. In phosphate limiting conditions (0.03 g L^−1^ K_2_HPO_4_) biomass formation was limited. The lack of biocatalyst led to a production of only 2.2 g L^−1^ polyol lipid in 10 days. To sum up, it was shown that under the selected conditions, 1.4 g L^−1^ K_2_HPO_4_ is sufficient to form the biomass required for complete glucose metabolization. Contrary to the results from the DoE, it is therefore not necessary to increase the K_2_HPO_4_ concentration to 6.3 g L^−1^ in order to achieve higher titers.

#### 3.3.4 Comparing C/N ratios—higher biomass is necessary to metabolize high amounts of glucose

In the two-level factorial design, C/N ratios of 50, 125, and 200 were investigated. With a *p*-value above 0.05, the C/N ratio was set as insignificant and was thus kept constant according to the center point of 125. However, in theory, higher amounts of nitrogen allow higher biomass, resulting in higher biocatalyst availability for polyol lipid production. During the optimization process, glucose concentration was increased to 208 g L^−1^, and due to a constant C/N ratio the NH_4_NO_3_ concentration automatically increased from 0.95 g L^−1^ to 1.91 g L^−1^. To investigate the C/N ratio’s influence on polyol lipid production, a one-factor-at-a-time experiment in MTPs using the optimized MLP medium with the final NH_4_NO_3_ concentration of 1.91 g L^−1^ and the initial 0.95 g L^−1^ were conducted. These concentrations relate to a doubled C/N ratio from 125 to 250 g_carbon_ g_nitrogen_^−1^. The results are shown in Figure 6B. With the higher nitrogen content and lower C/N ratio, after 216 h, a maximal polyol lipid titer of 41 g L^−1^ was reached, while using a lower nitrogen content, after 240 h, only 18 g L^−1^ was reached. Moreover, in the latter cultivation 114 g L^−1^ of glucose was left. In conclusion, the formed biomass using a C/N ratio of 250 g_carbon_ g_nitrogen_^−1^ could not metabolize the whole glucose into products within 10 days, resulting in lower polyol lipid titers. Contrary to the assumption from the DoE, the C/N ratio does have an influence, but not in the chosen borders. This finding aligns with Leathers et al. (2018) results in 2018. Higher C/N ratios translate to more carbon being available for secondary metabolite formation after the growth phase. Lower ratios lead to lower yields because carbon is first used for biomass formation (Gibbs and Seviour, 1996). Doshida et al. (1996) achieved only a Y_P/S_ of 0.14 g g^−1^ with a C/N ratio of 38. For lipid production using yeasts, an initial C/N ratio of 30–80 is recommended in the literature (Sitepu et al., 2014).

### 3.4 Transferring into scale: achieving a seamless transition from MTP to bioreactor scale

While MTP cultivations allow high-throughput investigation of cultivation conditions at low cost, bioreactor cultivations offer generation of precise online data and automated feeding controls. In bioprocess development, however, reproducibility is a well-known challenge when the process is transferred from a shaken to a stirred system (Büchs, 2001). For these reasons, the cultivation with the developed minimal medium was transferred from the 2 mL MTP scale into a 700 mL stirred-tank bioreactor cultivation. Further, the optimized minimal medium was compared with the synthetic starting medium. To enable a comparison between MTP and bioreactor, batch fermentations were performed as described in Chapter 2.4.4 without pH control. The process parameters are summarized in Table 7.

**TABLE 7:** Comparison of performance parameters using *A. pullulans* NRRL 62042: Comparison of performance parameters (maximal growth rate (µ_max_), polyol lipid, cell dry weight (CDW) after growth (48 h) and after 240 h, pullulan, product-to-substrate yield (Y_P/S_), product-to-biomass yield (Y_P/X_) after growth (48 h) and after 240 h, and space-time yield (STY)) between minimal liamocin production (MLP with 126 g L^−1^ glucose and 1 g L^−1^ K_2_HPO_4_) and optimized minimal liamocin production medium (oMLP with 208 g L^−1^ and 6.3 g L^−1^ K_2_HPO_4_) cultivating *A. pullulans* NRRL 62042 on MTP (24-well microtiter plates with 2 mL filling volume, 30°C, 300 rpm at 50 mm for 10 days, n = 3) and bioreactor (s vessel with 0.7 L filling volume, 30°C, 30% DO controlled with 300–1,200 rpm stirring rate, 0.5 vvm, n = 2) scale.

		MLP		Optimized MLP	
		MTP	Bioreactor	MTP	Bioreactor
µ_max_	[h^−1^]	0.12	0.11	-	0.18
c_polyol lipid_	[g_PL_ L^−1^]	30.8	28.7	48.1	41.3
c_CDW, 48 h_	[g_CDW_ L^−1^]	9.1	8.2	-	17.2
c_CDW, 240 h_	[g_CDW_ L^−1^]	12.9	9.4	-	27.0
c_pullulan_	[g_pullulan, max_ L^−1^]	9.3	27.1	-	50.5
Y_P/S_	[g_PL_ g_Glc_^−^^1^]	0.24	0.23	0.23	0.20
Y_P/X 48 h_	[g_PL_ g_CDW_^−1^]	3.6	3.6	-	2.4
Y_P/X 240 h_	[g_PL_ g_CDW_^−1^]	2.4	3.1	-	1.5
STY	[g_PL_ L^−1^ h^−1^]	0.13	0.12	0.20	0.19

**FIGURE 7:**
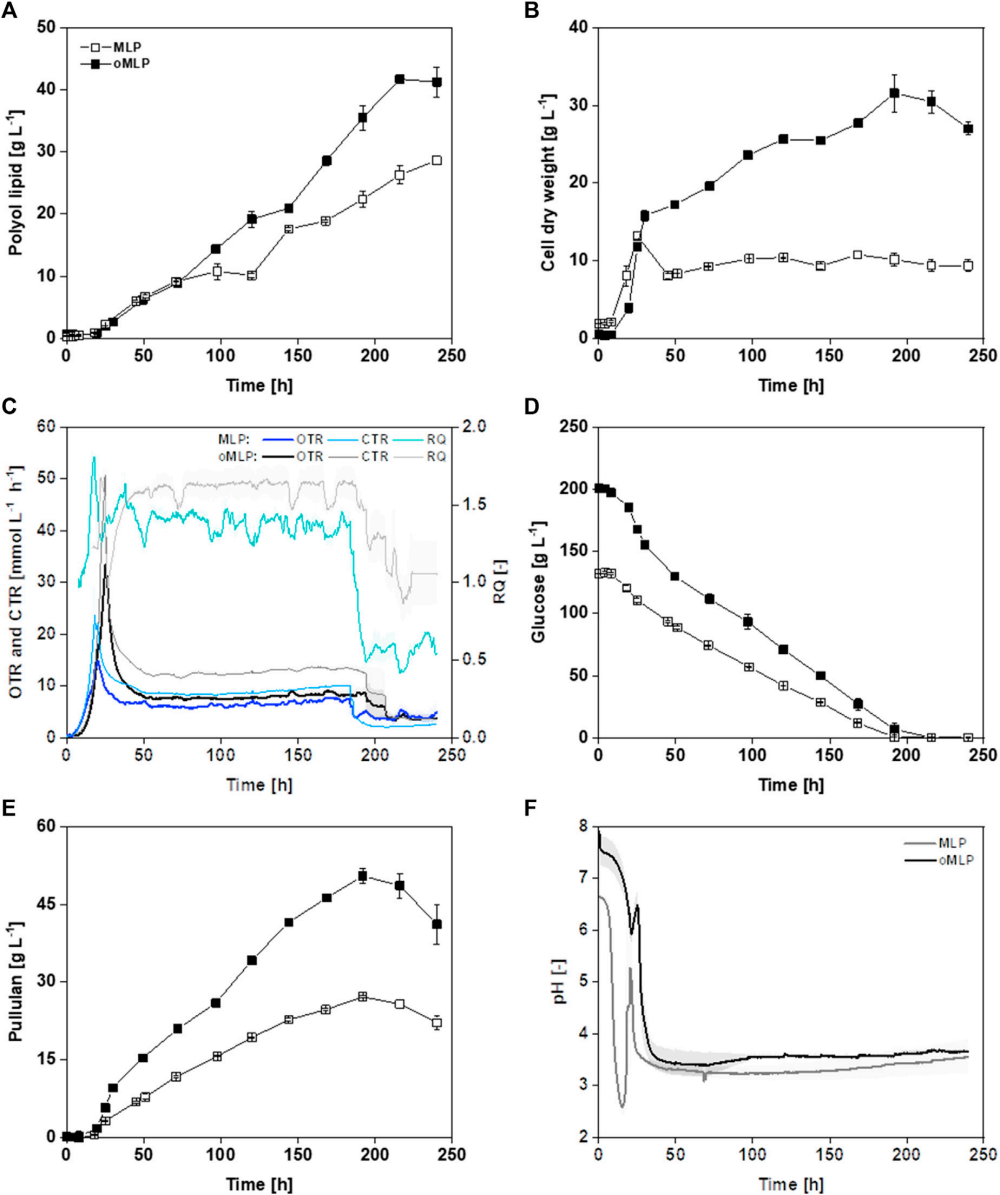
Bioreactor fermentation of *A. pullulans* NRRL 62042 with MLP and oMLP: Batch fermentation of minimal polyol lipid production (MLP with 126 g L^−1^ glucose, 0.95 g L^−1^ NH_4_NO_3_, and 1 g L^−1^ K_2_HPO_4_) and optimized minimal polyol lipid production (oMLP with 208 g L^−1^, 1.91 g L^−1^ NH_4_NO_3_, and 6.3 g L^−1^ K_2_HPO_4_) medium with *A. pullulans* NRRL 62042 (700 mL medium, 30°C, 30% DO-control by varying stirrer rate from 300-1,000 rpm, aeration with 100% air and 0.5 vvm, n = 2). Polyol lipid **(A)**, cell dry weight **(B)**, oxygen (OTR), and carbon dioxide (CTR) transfer rate and respiration quotient (RQ) **(C)**, glucose **(D)**, pullulan **(E)**, and pH **(F)** over time. The error bars show the deviation from the mean of biological duplicates.

In the following, MLP and oMLP medium were compared at bioreactor scale (Figure 7) During the optimization process, glucose concentration was increased from 126 to 208 g L^−1^, K_2_HPO_4_ concentration was increased from 1.0 to 6.3 g L^−1^, and NH_4_NO_3_ concentration increased from 0.95 to 1.91 g L^−1^. Overall, the polyol lipid titer in the bioreactor increased by almost 44% from 29 g L^−1^ (MLP) to 41 g L^−1^ (oMLP) (Figure 7A). Polyol lipid formation stagnated in both media, though at different timepoints, after 71 h (MLP) and 120 h (oMLP) and increased again later. Before the stagnation, the polyol lipid formation rate was 0.18–0.19 g L^−1^ h^−1^ for both. Medium optimization shortened the time of stagnation by approximately 24 h. Subsequently polyol lipid formation rate increased to 0.31 g L^-1^ h^−1^, while in MLP, it decreased to 0.12 g L^-1^ h^−1^. Using the oMLP medium, a higher final biomass of 27.0 g L^−1^ than in the MLP cultivation (13 g L^−1^) was reached (Figure 7B). Increasing carbon concentrations while maintaining a constant C/N ratio also implies proportionally rising nitrogen concentrations, leading to enhanced growth. The maximal growth rate was increased from 0.11 h^-1^ to 0.18 h^-1^. It should be noted that only a few samples were taken during the growth phase, and the growth rate could, therefore, be inaccurate. However, a faster growth using oMLP compared to MLP can be seen in Figure 7B. Figure 7C shows a peak in oxygen transfer rate (OTR) of 33 mmol L^−1^ h^−1^ (oMLP) and 17 mmol L^−1^ h^−1^ (MLP), followed by a constant value between 8 and 10 mmol L^−1^ h^−1^ until carbon depletion. This course suggests a substrate limitation except carbon (Anderlei and Büchs, 2001), which may be assigned to a nitrogen limitation based on CDW measurements and the high C/N ratio. The respiration quotient, calculated by dividing carbon dioxide by the oxygen transfer rate, was in the range of 1.5 after growth, which means that more CO_2_ is produced than O_2_ is consumed, indicating the production of a reduced product. Depending on the congener, the degree of reduction of polyol lipid is 5–5.2, so a respiration quotient greater than 1 could be attributed to polyol lipid production. The growth was terminated after approximately 50 h, and the following biomass increase might possibly be attributed to the formation of intracellular lipids. A renewed decrease in oxygen transfer rate suggests the consumption of glucose. After 240 h, the cultivation with the oMLP reached a biomass of 27 g L^−1^, whereas after 50 h, only 17 g L^−1^ was present. 10 g L^−1^ was thus probably due to intracellular storage lipid formation. The Y_P/X_ was 1.5 g g^−1^ and 2.4 g g^−1^, respectively. Figure 7D shows that the substrate decrease in the stationary phase is linear. The glucose depletion rate in the stationary phase before the optimization was 0.6 g L^−1^ h^−1^ and increased to 1.0 g L^−1^ h^−1^ after optimization. After the consumption of glucose, pullulan was degraded (Figure 7E). The highest pullulan concentrations were 27.1 g L^−1^ with the MLP and 46.1 g L^−1^ with the oMLP medium. The pullulan-to-substrate yield was 0.22 g g^−1^ for both. An increased glucose concentration led to the increased formation of storage substances such as pullulan, intracellular lipids, and polyol lipid. In both cultivations, the pH oscillated during the growth phase (Figure 7F). This was due to the metabolization of NH_4_NO_3_. Successively, the weak base (NH_4_^+^) and the weak acid (NO_3_^−^) were consumed. In addition, possibly released polymalate has a free acid group and thus might additionally lower the pH.

Comparing cultivations in MTPs and the bioreactor, growth rates, polyol lipid formation, Y_P/S_, Y_P/X_, and space-time yields (STY) were in a comparable range (Table 7). Regarding polyol lipid production, the upscaling had no effect when MLP was used and a slightly negative effect in the case of oMLP. It is striking that the switch from a shaken to a stirred system led to an almost threefold increase in the pullulan titer (9.3 g L^−1^ versus 27.1 g L^−1^). This effect has already been described in literature. Pullulan production can be influenced not only by the pH value but also by the agitation rate or energy input, whether positive or negative, depending on the strain. In a batch fermentation with *A. pullulans* ATTC9348, a tenfold increase in agitation rate led to a halving of the pullulan titer (Gibbs and Seviour, 1996). In contrast, in the fermentation of an *A. pullulans* strain that is not described in detail, the pullulan titer increased with increasing stirrer speed (McNeil and Kristiansen, 1987). In terms of biomass, comparing the cultivation in MTPs with the MLP medium in the bioreactor, nearly constant biomasses of 9.5–10 g L^−1^ were reached. A decrease in Y_P/X_ was probably due to increased pullulan and storage lipid production.

All in all, the developed and optimized minimal medium was successfully transferred from MTPs to the bioreactor with a scaling factor of 350. This resulted in 41 g L^−1^ polyol lipid, which is the highest polyol lipid titer reported with an *A. pullulans* wildtype strain using a laboratory-scale bioreactor.

## 4 Discussion

In recent decades, the chemical industry has become more efficient in providing cost-competitive products (Yu et al., 2019). The downsides are that many products are petroleum-based, produced in energy-intensive processes at high temperatures and pressures, and toxic solvents and reagents are used, resulting in pollution, greenhouse gases, and nonrecyclable or nondegradable by-products (Yu et al., 2019; Zimmerman et al., 2020). Green chemistry strives to avoid those downsides and has made great progress in the last years, aiming at circular processes based on renewable feedstocks, increasing atom efficiency, and using harmless chemicals, with the focus not only on maximizing function but also on minimizing hazards (Sheldon et al., 2007; Zimmerman et al., 2020; Sheldon and Brady, 2022; Sigmund et al., 2023). To date, competition with the chemical industry is challenging for the biotechnological sector, amongst other things because of using cost-intensive raw materials for biotechnological processes compared to cheap petroleum-based substrates, moreover in fully depreciated plants. Improving performance parameters by metabolic and process engineering, coupled with recruiting renewable and waste-based raw materials, is the key to success in competing with the chemical industry. Chen and Jiang (2018) summarized the goals for the next-generation industrial biotechnology to compete with the chemical industry. Among other things, parts of the next-generation industrial biotechnology are reducing energy consumption by applying unsterile process management, using one platform strain for multiple products, increasing product-to-substrate yields by removing or weakening competing pathways, and using mixed carbon sources from waste streams (Chen and Jiang, 2018). The characteristics known from the literature and the findings from this work suggest that *A. pullulans* may be a perfect fit for these ambitious goals.

Due to its polyextremotolerance and its ability to secrete enzymes such as lipase, xylanase, and cellulase, *A. pullulans* is predestined to metabolize cheap and environmentally friendly second-generation raw materials (Nagata et al., 1993; Leathers et al., 2016; Prasongsuk et al., 2018; Zhang et al., 2019). Previous studies identified pretreated wheat straw as potential carbon sources (Price et al., 2017). Li et al. (2021) successfully used waste streams from gluconate and xylonate production for polyol lipid production. With sodium gluconate mother liquor and xylonate mother liquor, a maximum polyol lipid titer of 28 g L^−1^ was achieved. The oMLP medium developed in this work lays the foundation for the targeted investigation of several carbon sources. For sophorolipid production, various waste or side streams such as soy molasses, coconut fatty acid residues, sugarcane molasses, crude soybean oil, waste cooking oil, or rice straw have already been used in recent years (Roelants et al., 2019). Moreover, for rhamnolipid production, starch-rich, frying oil, or oil processing waste could be used successfully (Mohanty et al., 2021). However, the advantage of low-cost substrates can be quickly nullified by poor efficiency and increased purification efforts. Besides carbon sources, up to 80% of production costs comprise product purification (Roelants et al., 2019). By using NH_4_NO_3_, costly yeast extract and peptone and the formation of melanin could be avoided in this work, eliminating one of such purification steps. Another important factor of the next-generation industrial biotechnology is energy consumption due to sterilization (Chen and Jiang, 2018). During the DoE, the adjustment of pH was omitted, resulting in an initial pH value of the optimized medium of around 8. During cultivation, the pH dropped to 3. The wide pH range reflects the robustness of *A. pullulans* and can help avoid the need for sterilization. Furthermore, extreme pH values reduce the risk of contamination during continuous operations and enables semi-sterile feeding of industrial feedstocks (Li et al., 2014; Ernst et al., 2024). Besides advantages in energy consumption, the pH value can also positively affect production. In 2012, Cao et al. cultivated *A. pullulans* ipe-1 in a bioreactor at constant pH values (Cao et al., 2013). The highest pH value of 7 coincided with the poorest growth. Therefore, an initial pH adjustment could enhance cell growth. Furthermore, Saur et al. (2019) achieved increased productivity in polyol lipid production through a pH shift from 6.5 to 3.5 during fermentation.

A comprehensive examination of processes always depends on reliable analytical methods. The methods used in the literature and this study exhibit certain sources of error that must be considered and, which we briefly explain here. Without energy input, polyol lipids sediment rapidly in the culture broth, complicating homogenous sampling from shake flasks. By employing MTPs and harvesting the entire well, coupled with additional rinsing of the well, homogeneous sampling can be ensured. For the polyol lipid determination, the aqueous phase of the culture broth is initially separated through centrifugation. The precipitate includes the cell pellet and the polyol lipid. To separate the polyol lipid from the cells, it is dissolved in a solvent and separated from the cellular components through centrifugation. A final evaporation step separates the polyol lipid from the solvent, and the concentration can be determined gravimetrically. However, in addition to polyol lipids, remaining water, soluble cell components, and intracellular lipids from the destroyed cells are also weighed. As a result, gravimetrically determined polyol lipid titers are likely overestimated. Furthermore, comparing results between different strains and publications is hindered. An obvious solution to these challenges is using a quantitative analytical method like chromatography. Initial steps towards high-performance liquid chromatography (HPLC) analysis have been taken, allowing, at least, the exclusion of water and quantification without external standards by using an inverse gradient as described for rhamnolipids (Behrens et al., 2016; Scholz et al., 2020). However, the precise separation of individual congeners remains challenging to date (Scholz et al., 2020). Adequate HPLC analysis would enable precise product quantification and, additionally, facilitate the investigation of the influence of cultivation conditions on congener distribution. Overall, *A. pullulans* already fulfills important criteria to become part of next-generation industrial biotechnology (Chen and Jiang, 2018). However, there is still much potential for optimization regarding process design and strain engineering. Furthermore, in order to create comparability and to investigate possible influencing factors, a standardized HPLC method is required that enables the identification and quantification of the individual congeners.

## 5 Conclusion

In this work, a minimal medium was developed for polyol lipid production with *A. pullulans* NRRL 62042. The production performance achieved was similar to the currently best performing medium containing complex components. Subsequently, the titer was increased by 56% to 48 g L^−1^ using a DoE approach. Currently, this is the highest biosurfactant titer achieved using an *A. pullulans* wild-type strain. Furthermore, the space-time yield was increased from 0.13 to 0.20 g L^−1^ h^−1^. This was followed by successfully transferring the cultivation from 2 mL MTP into 0.7 L bioreactor scale. Overall, this work demonstrated the potential of *A. pullulans* for large-scale biosurfactant production. While it is already possible to achieve high titers and Y_P/S_ using the wild type, further optimization can be achieved by strain engineering and process design. The developed minimal medium allows the targeted investigation of alternative carbon sources for economic process optimization and the investigation of the metabolic pathway to identify targets for genetic optimization.

## Data Availability

The original contributions presented in the study are included in the article/Supplementary Material, further inquiries can be directed to the corresponding author.
