# Supplementary material for: DoE-based medium optimization for improved biosurfactant production with Aureobasidium pullulans
Source: Front Bioeng Biotechnol. 2024 Mar 6;12:1379707. doi: 10.3389/fbioe.2024.1379707 (PMC10953688; doi:10.3389/fbioe.2024.1379707)
Supplement: Supplementary file 1 [file DataSheet1.pdf]

Supplementary Material

DoE-based medium optimization for improved biosurfactant production with *Aureobasidium pullulans*

Frederick Haala[1], Marie R. E. Dielentheis-Frenken[1], Friedrich M. Brandt[1], Tobias Karmainski[1], Lars M. Blank[1], Till Tiso[1*]

[1]Institute of Applied Microbiology, RWTH Aachen University, Aachen, Germany

*** Correspondence:**
Corresponding Author
till.tiso@rwth-aachen.de

1 Supplementary

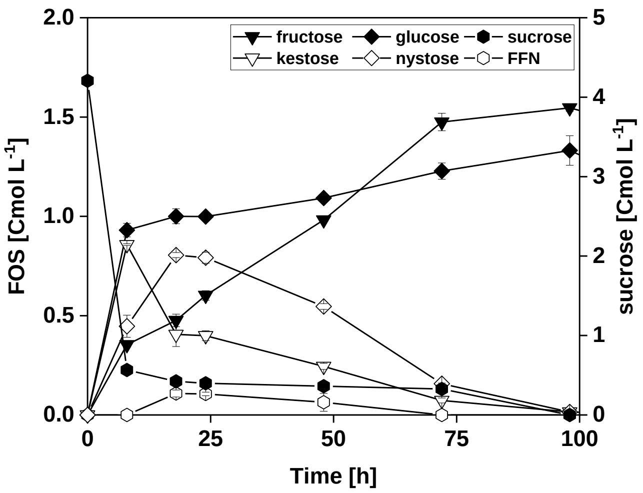

Figure S1: Fructooligosaccharide (FOS) formation and depletion using sucrose as carbon source: Concentration of sucrose, kestose, nystose, and fructofuranosylnystose (FFN) and their monomers glucose and fructose in the first 100 hours. Cultivations were performed with *A. pullulans* NRRL 62042 in 24 deep-well microtiter plates with 2 mL filling volume, 30 °C, and 300 rpm. Data show the mean of biological triplicates with ± standard deviation.

Table S1: Two-level factorial design plan of experiments with factors C/N ratio, vitamin (vit.) solution, trace-element (TE) solution, K_2HPO_4, $MgSO_4 \times 7\ H_2O$ $MgSO_4$, NaCl, Glucose, and the response polyol lipid titer.

**Run**	**C:N ratio**	**Vit.-Sol.**	**TE-Sol.**	**K_2HPO_4**	**$MgSO_4 \times 7\ H_2O$**	**NaCl**	**Glucose**	**Polyol lipid**
	[$g_C\ g_N^{-1}$]	[ml L^{-1}]	[ml L^{-1}]	[g L^{-1}]	[g L^{-1}]	[g L^{-1}]	[g L^{-1}]	[g L^{-1}]
**1**	200	0.5	5	0.5	2	0.5	150	28
**2**	50	2	15	2	2	0.5	50	7.95
**3**	50	0.5	15	0.5	0.5	0.5	50	9.45
**4**	50	2	5	2	2	2	150	27.05
**5**	50	2	15	0.5	0.5	2	150	18
**6**	200	2	5	2	2	0.5	50	5.75
**7**	200	2	15	2	0.5	2	50	6.6
**8**	50	2	15	0.5	2	2	50	8.3
**9**	50	2	5	0.5	2	0.5	150	21.85
**10**	200	2	15	2	2	2	150	27.9
**11**	200	0.5	5	0.5	0.5	0.5	50	9.5
**12**	50	0.5	5	2	2	0.5	50	8
**13**	50	2	5	0.5	0.5	0.5	50	7.95
**14**	200	2	5	2	0.5	0.5	150	32.65
**15**	50	2	15	2	0.5	0.5	150	25.95
**16**	200	0.5	15	0.5	0.5	2	150	25.7
**17**	50	2	5	2	0.5	2	50	7.1
**18**	50	0.5	15	2	2	2	150	29.75
**19**	200	0.5	5	2	0.5	2	50	8.85
**20**	125	1.25	10	1.25	1.25	1.25	100	22.9
**21**	50	0.5	5	2	0.5	0.5	150	31.6
**22**	200	2	5	0.5	2	2	50	9.4

**23**	200	2	15	0.5	2	0.5	150	29.35
**24**	200	2	15	0.5	0.5	0.5	50	7.3
**25**	200	0.5	15	2	2	0.5	50	7.9
**26**	200	2	5	0.5	0.5	2	150	25.85
**27**	125	1.25	10	1.25	1.25	1.25	100	20.5
**28**	50	0.5	15	2	0.5	2	50	6.65
**29**	125	1.25	10	1.25	1.25	1.25	100	20.7
**30**	50	0.5	5	0.5	0.5	2	150	23.8
**31**	50	0.5	15	0.5	2	0.5	150	19.1
**32**	200	0.5	15	2	0.5	0.5	150	29.3
**33**	200	0.5	15	0.5	2	2	50	8.7
**34**	125	1.25	10	1.25	1.25	1.25	100	20.05
**35**	50	0.5	5	0.5	2	2	50	8
**36**	200	0.5	5	2	2	2	150	26.15

Table S2 ANOVA analysis from two-level factorial design with the factors carbon-to-nitrogen ratio C/N ($g_{carbon}\ g_{nitrogen}^{-1}$), vitamin and trace-element solution, K_2HPO_4, $MgSO_4 \times 7\ H_2O$, NaCl, and glucose.

Factor	Sum of squares	Degrees of freedom	Mean square	F-value	p-value
**Model**	2,981.05	26	114.66	17.50	< 0.0001
**C/N ratio (A)**	25.20	1	25.20	3.85	0.0815
**Vit.-solution (B)**	4.13	1	4.13	0.6308	0.4475
**TE-solution (C)**	5.78	1	5.78	0.8823	0.3721
**K_2HPO_4 (D)**	26.10	1	26.10	3.98	0.0771
**$MgSO_4 \times 7$ H2O (E)**	0.3003	1	0.3003	0.0458	0.8352
**NaCl**	5.95	1	5.95	0.9084	0.3654
**Glucose (F)**	2,712.16	1	2,712.16	413.98	< 0.0001
**AB**	5.20	1	5.20	0.7938	0.3962
**AC**	1.45	1	1.45	0.2206	0.6498

**AD**	21.62	1	21.62	3.30	0.1027
**AE**	0.1378	1	0.1378	0.0210	0.8879
**AF**	1.71	1	1.71	0.2612	0.6216
**AG**	23.12	1	23.12	3.53	0.0930
**BD**	0.2813	1	0.2813	0.0429	0.8405
**BE**	7.41	1	7.41	1.13	0.3152
**BF**	0.3403	1	0.3403	0.0519	0.8248
**CD**	0.3403	1	0.3403	0.0519	0.8248
**CE**	16.68	1	16.68	2.55	0.1451
**CF**	0.6050	1	0.6050	0.0923	0.7681
**CG**	3.25	1	3.25	0.4963	0.4990
**DE**	5.61	1	5.61	0.8565	0.3789
**DG**	73.51	1	73.51	11.22	0.0085
**ADE**	12.75	1	12.75	1.95	0.1964
**ADG**	16.10	1	16.10	2.46	0.1514
**BDE**	5.70	1	5.70	0.8693	0.3755
**CDE**	5.61	1	5.61	0.8565	0.3789
**Lack of Fit**	54.12	6		5.58	0.0932
**Pure Error**	4.85	3			

Table S3: Central composite design plan of experiments with factors glucose and K_2HPO_4, and the response polyol lipid titer and $Y_{P/S}$.

**Run**	**Glucose**	**K_2HPO_4**	**Polyol lipid**	**$Y_{P/S}$**
	g L^{-1}	g L^{-1}	g L^{-1}	g g^{-1}
**1**	120.00	8.00	24.80	0.21
**2**	300.00	1.40	27.95	0.09
**3**	120.00	1.40	29.80	0.25
**4**	210.00	4.70	45.80	0.22

**5**	300.00	8.00	35.45	0.12
**6**	210.00	4.70	45.70	0.22
**7**	120.00	1.40	27.15	0.23
**8**	300.00	1.40	30.75	0.10
**9**	120.00	8.00	23.15	0.19
**10**	120.00	8.00	21.15	0.18
**11**	300.00	1.40	32.80	0.11
**12**	120.00	8.00	25.70	0.21
**13**	120.00	1.40	25.45	0.21
**14**	120.00	1.40	24.75	0.21
**15**	300.00	8.00	31.20	0.10
**16**	300.00	1.40	25.75	0.09
**17**	120.00	8.00	20.45	0.17
**18**	300.00	8.00	39.90	0.13
**19**	300.00	8.00	41.50	0.14
**20**	300.00	1.40	31.20	0.10
**21**	210.00	4.70	36.15	0.17
**22**	120.00	1.40	26.20	0.22
**23**	300.00	8.00	37.30	0.12
**24**	210.00	4.70	46.70	0.22
**25**	210.00	4.70	50.10	0.24
**26**	82.72	4.70	18.85	0.23
**27**	82.72	4.70	18.00	0.22
**28**	210.00	0.03	2.20	0.01
**29**	210.00	9.37	41.75	0.20
**30**	210.00	9.37	44.55	0.21
**31**	82.72	4.70	17.80	0.22
**32**	337.28	4.70	36.85	0.11
**33**	337.28	4.70	35.15	0.10

**34**	82.72	4.70	18.65	0.23
**35**	210.00	4.70	49.70	0.24
**36**	210.00	9.37	44.20	0.21
**37**	210.00	0.03	3.15	0.01
**38**	210.00	0.03	3.15	0.01
**39**	337.28	4.70	36.90	0.11
**40**	337.28	4.70	35.85	0.11
**41**	210.00	9.37	39.50	0.19
**42**	82.72	4.70	19.10	0.23
**43**	210.00	0.03	2.95	0.01
**44**	210.00	9.37	42.80	0.20
**45**	337.28	4.70	36.85	0.11
**46**	210.00	4.70	47.00	0.22
**47**	210.00	0.03	2.65	0.01
**48**	210.00	4.70	50.00	0.24
